# Awareness and Perceptions of School Children about Female Feticide in Urban Ludhiana

**DOI:** 10.4103/0970-0218.66873

**Published:** 2010-04

**Authors:** Anurag Chaudhary, Mahesh Satija, Sarit Sharma, GPI Singh, R K Soni, R K Sachar

**Affiliations:** Department of Community Medicine, Dayanand Medical College and Hospital, Ludhiana, Punjab, India

**Keywords:** Awareness, female feticide, girl child, school children, son preference

## Abstract

**Background:**

Although the Indian girl child’s position is precarious throughout the country, she remains the most vulnerable in Punjab.

**Objectives:**

To assess the awareness and perceptions of school children regarding female feticide.

**Study Design:**

Crosssectional study.

**Materials and Methods:**

The study involved collection of information regarding knowledge and perception of school students about female feticide using multiple choice questionnaire. A total of 527 students between the age group of 11-18 years of various schools of district Ludhiana, Punjab were the study subjects. They had come to participate in the poster competition on organ donation (SAARC Transplant games), organized by Department of Community Medicine, D.M.C and H, Ludhiana.

**Results:**

Out of total 527 students, 97.9% were aware of female feticide. Main source of information was TV (56%), followed by newspaper (33%). Majority of the students (65.2 %) felt that discrimination between boys and girls is prevalent in the society. Regarding perception of school students for curbing this social evil, 37.8% school students were of the view that awareness among the masses is the solution to stop this practice, while 25% of the students responded that equal status to girls will stop this practice of female feticide.

**Conclusions:**

The school students had optimum level of awareness about female feticide and almost all of them strongly felt that this harmful practice should be stopped altogether.

## Introduction

It is a matter of grave concern that girl child continues to be insecure and vulnerable in the state of Punjab. As per census 2001, the sex ratio (0-6 years) in Punjab declined from 875 to 793 despite the fact that the law (PC-PNDT ACT) specifically prohibits pre-natal sex determination. Therefore, the need of the hour is to stress upon other avenues/alternatives that can strengthen the law and can bring about desired social change. One such alternative is by increasing the awareness in the community about female feticide, so that the people can identify it as a social problem and further try to curb female feticide. Community awareness against female feticide can be increased by developing a work force of educated boys and girls who can spread the awareness against the social evil. School education given to children is often termed as ‘social vaccine’ as school children act as powerful medium in dissemination of information in the society and therefore act as an effective preventive tool.(1)

The Planning Commission of India estimates that as of March 2000, adolescents aged 10–19 comprised 23% of the Indian population, i.e. almost 230 million. Such a large group represents a major human resource that can and must contribute to the overall development of the country.(2) It is this sizeable number of adolescents who would be the future mothers and fathers, who would either be victims of the social evils or be the ones who would end up perpetrating social evils. We must prevent this from happening. Rather than allowing them from becoming either ‘victims’ or ‘perpetrators’ or ‘silent sufferers’, we need to induct these students in our fight against social evil such as sex-determination and female feticide. They are at a stage where they could be moulded and influenced.(3) With this background, the present study was done with the following objectives: (1) To assess the awareness of school children regarding female feticide; (2) To know the perceptions of school children about female feticide; and (3) To give recommendations for increasing the awareness among adolescents against this social evil.

## Materials and Methods

The present study was conducted by the Department of Community Medicine, Dayanand Medical College and Hospital, Ludhiana. A total of 527 students studying in 35 schools of Ludhiana district were the study subjects. Out of 527 school students, 464 were studying in private schools and 63 students were studying in government schools respectively. Age of school students ranged from 11 to 18 years. They had come to participate in the poster competition on organ donation (SAARC Transplant games), organised by the Department of Community Medicine, D.M.C and H, Ludhiana. Information was collected by administering a pretested questionnaire to the students that had multiple choice questions and one open ended question. The questionnaire was also translated into vernacular language.

After taking the consent of the head teacher, the purpose of the study was explained to the students. The students were asked to fill the questionnaire in the presence of investigators and class teacher. They were not permitted to communicate with each other. Data were entered and analyzed by means of simple comparisons and proportions. Investigators read the open-ended question in each questionnaire and themes were generated.

## Results

The study subjects consisted of 154 male and 362 female students, respectively (11 subjects did not specify their sex) [Table 1].

**Table 1:** Awareness about female feticide

Variable	Male	Female	Total
What is female feticide (correct	152 (98.7)	354 (98.6)	516 (97.9)
response)			
Female feticide is harmful for	150 (100)	343 (97.7)	493 (98.4)
society (yes)			
Practice of female feticide	149 (99.3)	356 (99.4)	505 (99.4)
should be stopped (yes)			
Whether equal opportunities	128 (83.10)	296 (82)	424 (82.3)
should be given to girls (yes)			
Whether educated about	135 (91.2)	312 (87.2)	447 (86.5)
female feticide in school (yes)			

Figures in parenthesis are percentages

Study subjects showed high level of awareness about female feticide, 97.9% of the students knew correctly about female feticide. Male and female students had almost equal level of awareness (98.7% and 98.6% respectively). Majority of the students (98.4%) agreed to the fact that female feticide is harmful for society. They were also of the opinion that this practice should be stopped (99.4%). It was interesting to observe that in response to question ‘whether equal opportunities are given to girls as compared to boys’, 82.3% of the students gave response in affirmation. In the similar context when the students were asked about their own experience regarding discrimination between boys and girls, a majority of them (65.2%) had felt the discrimination between boys and girls in the society, in the family (9.5%) and in the school (2.9%). Increased awareness of school students about female feticide can be attributed to education given to them in schools, 86.5% of the students were given education about female feticide in their respective schools. Regarding source of information [Table 2], besides being taught in schools, 56% of the students got information about female feticide from television, 33.2% from newspaper, 6.3% and 4.5% got information from family and friends, respectively.

**Table 2:** Source of information

Source of information	*N*=509
Television	285 (56)
Newspapers	169 (33.2)
Family	32 (6.3)
Friends	23 (4.5)

Figures in parenthesis are percentages

Perceptions of students regarding reasons for son preference and no preference for girl child were generated through multiple choice questions [Table 3]. Maximum responses (45.7%) were for the choice that sons are preferred because they carry name of family and they take care of their parents in old age, followed by 44.7% responses for the choice that they carry the name of the family alone. When asked why girl child is not preferred, maximum responses (59.1%) were received for all the choices combined namely, burden on family, dowry and girls cannot take care of their parents in old age, this was followed by 22.1% responses for dowry as the main reason for not preferring girls in the family.

**Table 3:** Perceptions of students about male preference

Choices	Male	Female	Total
Why male child is preferred?	*N*=153	*N*=359	*N*=512
1. Carries name of family	52 (34)	177 (49.3)	229 (44.7)
2. Takes care of parents	11 (7.2)	32 (8.90)	43 (8.4)
3. Boys are more intelligent	2 (1.3)	4 (1.10)	6 (1.2)
4. Both 1 and 2	88 (57.50)	146 (40.70)	234 (45.7)
Why girl child is not preferred?	*N*=154	*N*=352	*N*=506
1. Burden on family	18 (11.70)	53 (15.10)	71 (14.0)
2. Dowry	35 (22.70)	77 (21.90)	112 (22.1)
3. Cannot take care of parents	7 (4.50)	17 (4.80)	24 (4.7)
4. All of the above	94 (61.00)	205 (58.20)	299 (59.1)

Figures in parenthesis are percentages

Perceptions of students regarding family composition and size can help us to know their behavior in coming future. When asked about ideal family composition, 99.4% of the students chose for the response, one boy and one girl. Regarding ideal family size, 94.9% responded for two children. It was an interesting observation that 4.3% of the students were in favor of only one child [Table 4].

**Table 4:** Perceptions of students regarding family composition and size

Family composition and size	Male (%)	Female (%)	Total (%)
Ideal family composition	*N*=152	*N*=361	*N*=513
One boy one girl	151 (99.3)	359 (99.4)	510 (99.4)
Only boys	1 (0.70)	0 (0.0)	1 (0.2)
Only girls	0 (0.0)	2 (0.6)	2 (0.4)
Ideal family size	*N*=153	*N*=360	*N*=513
Only one child	10 (6.5)	12 (3.3)	22 (4.3)
Two children	142 (92.8)	345 (95.8)	487 (94.9)
More than two children	1 (0.7)	3 (0.8)	4 (0.8)

## Themes arising

On analysis of one open ended question in the questionnaire “How this social evil can be stopped?” A variety of responses were obtained as all the students answered (except for few students) and following themes were generated.

## By increasing awareness in the society and parents (37.8 %)

"We can stop female feticide by doing functions, by rallies, by telling our parents, grandparents and neighbors. This can stop female feticide," a 14-year male student told.

## By giving equal status to girls (25.0%)

"First of all parents should be strong. At present girl is pride of the nation. The abortion should be strictly stopped. The girls should be given equal posts by the Government as well as corporations. The girls themselves should start campaign against this evil discrimination," a 15-year female student told.

Similarly other themes were generated as:

- By giving punishment to people and doctors involved (10.6%)
- By giving equal opportunities to girls (10.0%)
- By enforcing strict law (8.0%)
- By eliminating dowry system (5.7%)
- By enforcing ban on prenatal sex determination (5.1%)

## Discussion

The awareness about female feticide was found to be considerably high in the study subjects and 99.4% of these adolescents were of the opinion that this practice should be stopped. However, in a study done by Walia(4) among adult population in three districts of Punjab having low child sex ratio i.e. Ludhiana, Bhatinda and Ferozepur, though the awareness regarding female feticide was found to be very high, yet majority of the respondents approved of this heinous act.

In the present study, 44.7% of the school students cited carrying the name of family as the main reason for son preference whereas another 45.7% were of the view that in addition to carrying the family name, the sons also take care of their parents. A study conducted by MOHFW(5) in 2002 among 530 adult respondents in Delhi also observed that the reasons for son preference were that they carry on the family name and inherit property; they are providers in old age and perform the last rites.

Majority of the study subjects (59.1%) were of the opinion that girl child is not preferred as the girls are burden on the family, dowry system and girls cannot take care of their parents, while another 22.1% responses were in favor of dowry as the only reason for not preferring the girl child. As per study done by NIPCCD(6) in 2008 in Delhi and Haryana, dowry was perceived as the main reason for not preferring the girl child by the respondents.

Majority of the students perceived two children (one boy and one girl) as the ideal family composition as well as the ideal family size. Similar findings were observed among female respondents in the NIPCCD study.(6)

## Conclusion

This study reveals that the school students had optimum level of awareness about female feticide and almost all of them strongly felt that this harmful practice should be stopped altogether. Analysis of the themes generated gave an insight into the level of understanding of these adolescents toward this social evil as they touched upon all the major possible strategies for eliminating this practice.

As school students are important stakeholders in elimination of the practice of female feticide, it is recommended that these adolescents should be equipped with ample amount of knowledge so that they can act as change mediators in the society.
